# Supplementary material for: miRNA-214-3p stimulates carcinogen-induced mammary epithelial cell apoptosis in mammary cancer-resistant species
Source: Commun Biol. 2023 Oct 3;6:1006. doi: 10.1038/s42003-023-05370-4 (PMC10547694; doi:10.1038/s42003-023-05370-4)
Supplement: Supplementary file 2 — Supplementary Information [file 42003_2023_5370_MOESM2_ESM.pdf]

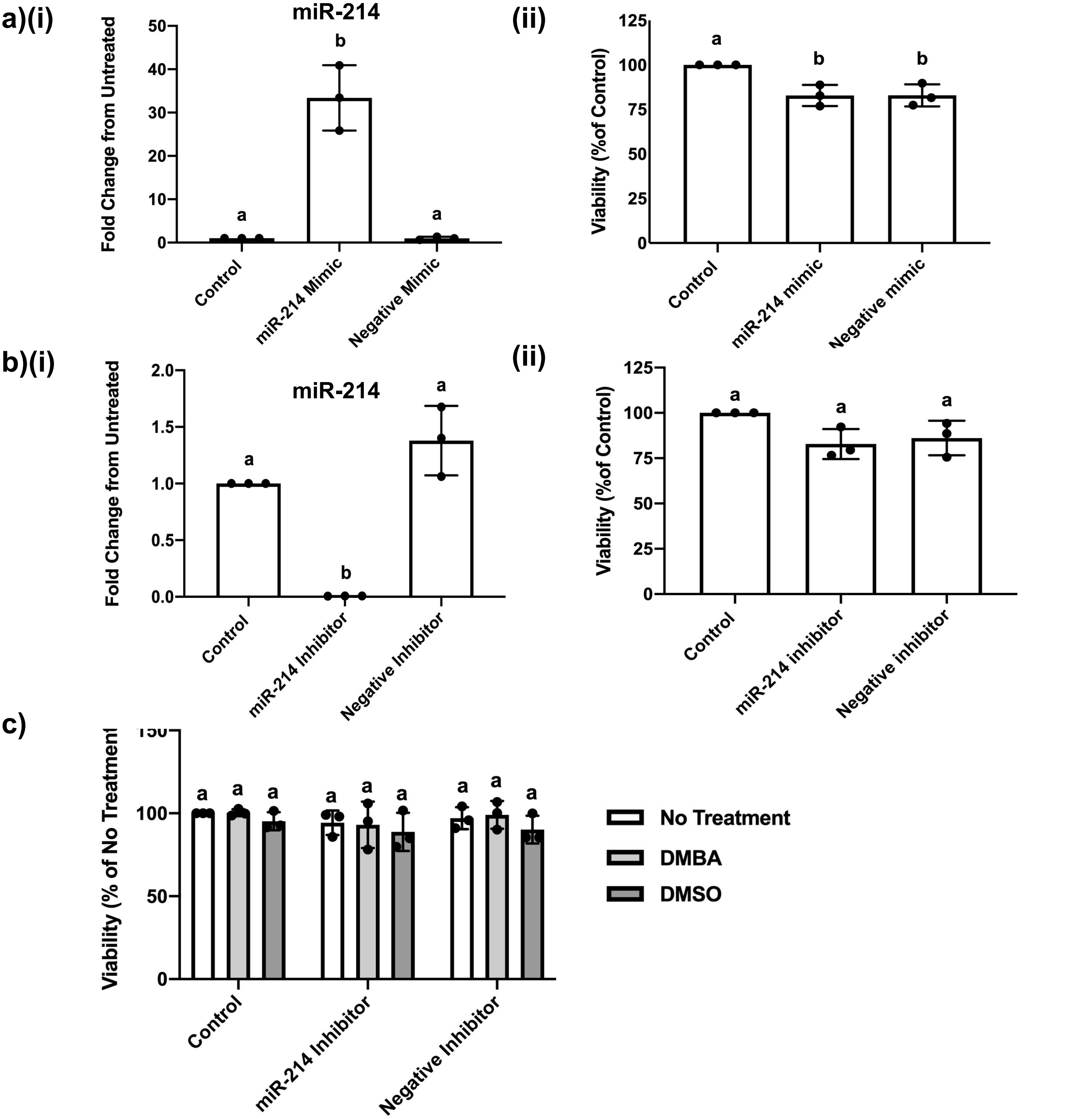

Supplementary Figure 1. MicroRNA-214 (miR-214) expression can be altered in equine and canine mammosphere-derived epithelial cells (MDECs) and inhibition of miR-214 in canine MDEC does not promote 7, 12-Dimethylbenz(a)anthracene (DMBA)-induced apoptosis. a) Quantitative reverse transcription-polymerase chain reaction (qRT-PCR) analysis of **(i)** miR-214 expression in and **(ii)** cell viability of equine MDECs that were untransfected, transfected with a miR-214 mimic, or transfected with a negative miRNA mimic. **b)** qRT-PCR analysis of **(i)** miR-214 expression in and **(ii)** cell viability of canine MDECs that were untransfected, transfected with a miR-214 inhibitor, or transfected with a negative miRNA inhibitor. **c)** Viability of canine MDECs that were untransfected, transfected with a miR-214 inhibitor, or transfected with a negative miRNA inhibitor, followed by no treatment, treatment with 5 μM DMBA, or treatment with the vehicle control dimethlysulfoxide (DMSO). n = 3. Error bars show standard deviations. Different letters above bars indicate statistically significant differences. $P < 0.05$.

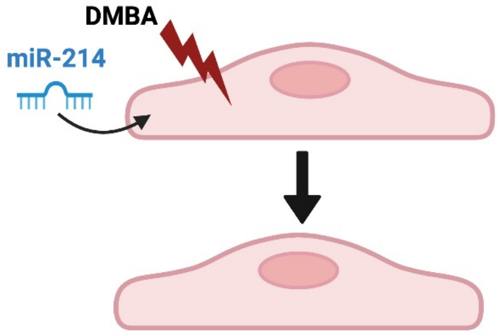

↓ *UNC5A*		↓ *MAPK1*		↓ *NFKB1*	
**Upregulated Targets**	**Downregulated Targets**	**Upregulated Targets**	**Downregulated Targets**	**Upregulated Targets**	**Downregulated Targets**
CDKN1A	*ABCB1*	*BAG2*	*CAD*	*EDN1*	*ABCB1*
CDKN2B	*BRCA1*	*CDKN1A*	*CCNB1*	*F3*	*BARD1*
JUN	*BRCA2*	*SRF*	*CCNE1*	*HES1*	*BRCA2*
MYC	*CCNB1*		*CDK2*	*HOXA9*	*CCNB1*
SIAH1	*CD82*		*CHEK2*	*MGMT*	*CCNE2*
TP5313	*CDK1*		*EZH2*	*MMP9*	*CCNF*
	CDK2		*FOXM1*	*PTGER4*	*CSF1*
	CDKN1A		*PCNA*	*PTGFR*	*CSF2*
	CHEK2		*PGK1*	*SNAI1*	*CXCL2*
	CRYAB		*PRDX3*	*TGFB1I1*	*CXCL8*
	E2F1		*ST3GAL1*	*TIMP1*	*E2F1*
	E2F7		*UBE2C*	*TRAF2*	*ERAP1*
	EZH2			*YY1*	*GCLC*
	FOXM1				*GSTP1*
	GSTP1				*ITGAX*
	HSP90AB1				*MMP14*
	IGF1R				*NCAM1*
	IGFBP3				*NFKB1*
	MAP4				*PLAU*
	MCM7				*PLAUR*
	MDM2				*PTGS2*
	NFKB1				*SQSTM1*
	POLD1				*TAPBP*
	PRC1				*TF*
	RECQL4				
	SLC2A1				
	STAT1				
	UHRF1				

Supplementary Figure 2. Transfection with a microRNA-214 (miR-214)-mimic and treatment with 7, 12- Dimethylbenz(a)anthracene (DMBA) leads to downregulation of unc-5 netrin receptor A *(UNC5A)*, mitogen-activated protein kinase 1 *(MAPK)1*, and nuclear factor kappa beta 1 *(NFKB1)* in equine mammosphere-derived epithelial cells (MDECs). Up and downregulation of some downstream targets of these 3 transcripts are listed. Images created with BioRender.com.

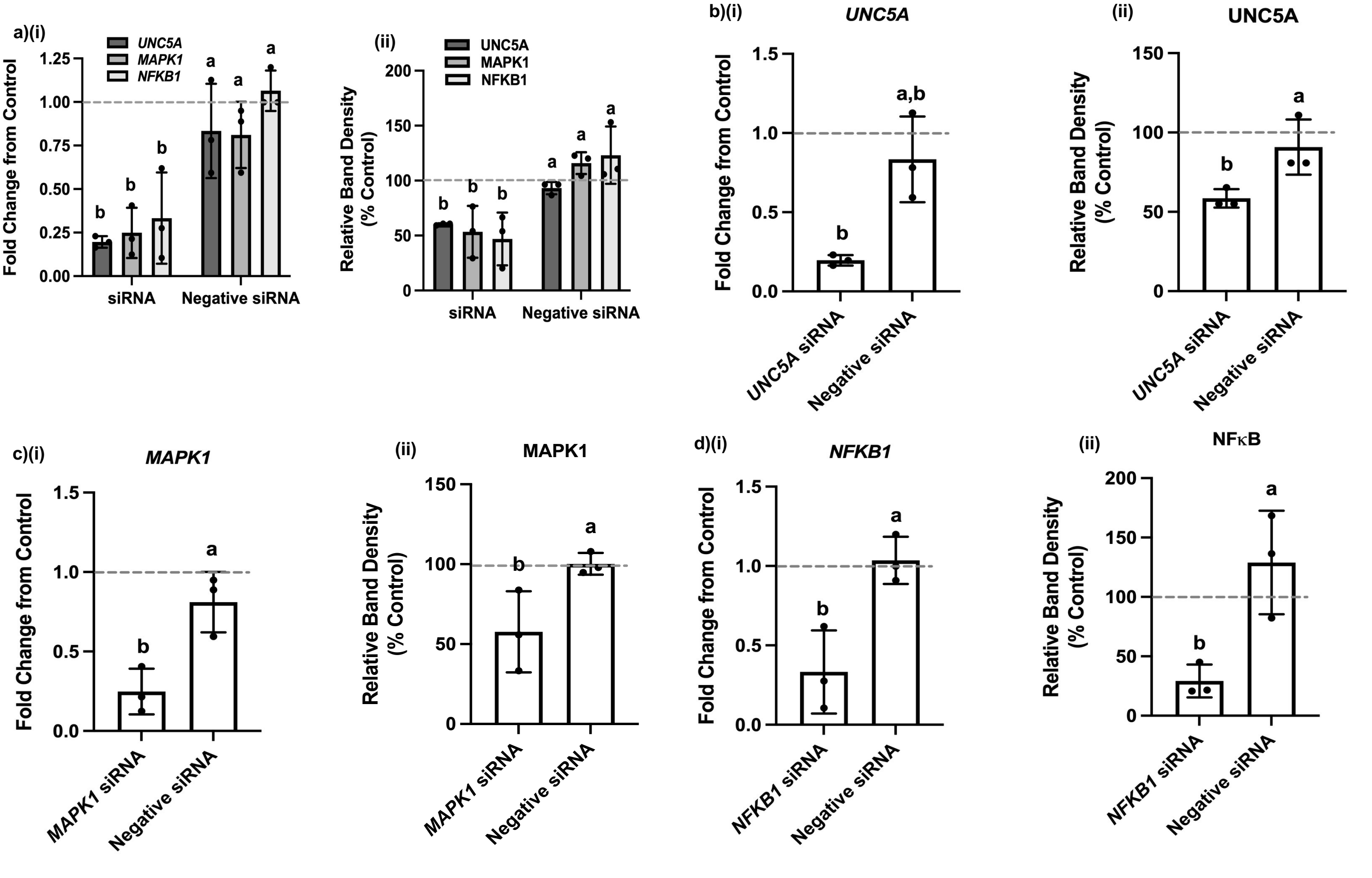

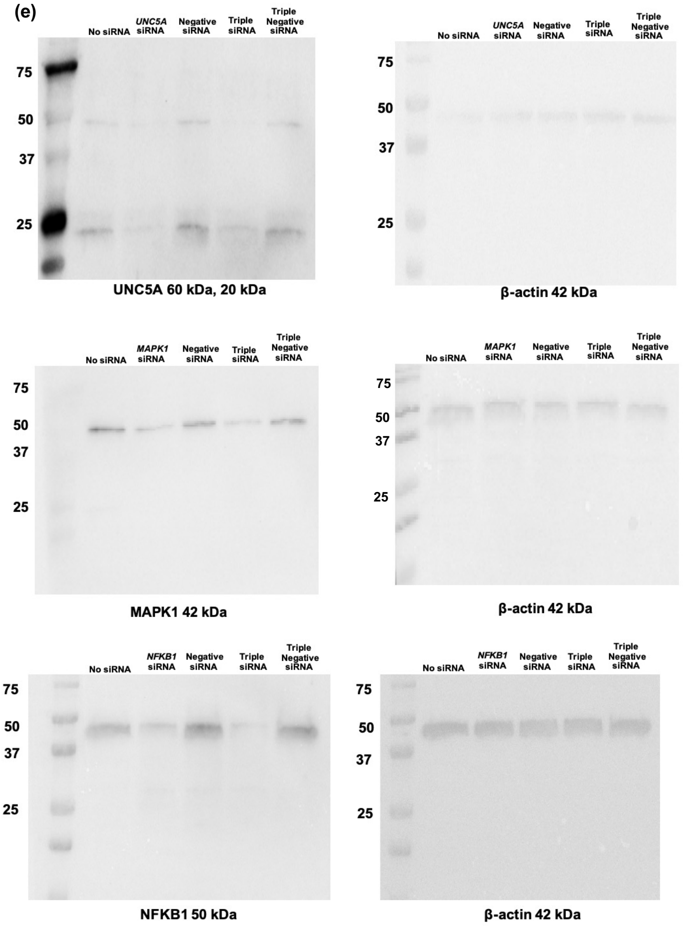

Supplementary Figure 3. Small-interfering RNA (siRNA) were used to knock down the expression of unc-5 netrin receptor A *(UNC5A)*, mitogen-activated protein kinase 1 *(MAPK)1*, and nuclear factor kappa beta 1 *(NFKB1)* in equine mammosphere-derived epithelial cells (MDECs). a) Quantitative reverse transcription-polymerase chain reaction (qRT-PCR) analysis **(i)** and Western blot analysis **(ii)** of *UNC5A*/UNC5A*,* *MAPK1*/ MAPK1 and *NFKB1*/NF-κB expression in equine MDECs transfected with a *UNC5A, MAPK1* and *NFKB1* siRNA cocktail or negative siRNA at the same concentration. **b), c), d)** qRT-PCR analysis **(i)** and Western blot analysis **(ii)** of either *UNC5A*/UNC5A **(b)**, *MAPK1*/ MAPK1 **(c)** or *NFKB1*/NF-κB **(d)** expression in equine MDECs transfected with either *UNC5A, MAPK1*, *NFKB1,* or negative siRNA. **e)** Representative images of Western blots for UNC5A, MAPK1, NFKB1, and reference protein *β*-actin. Error bars show standard deviations. Different letters above bars indicate statistically significant differences. $P < 0.05$.

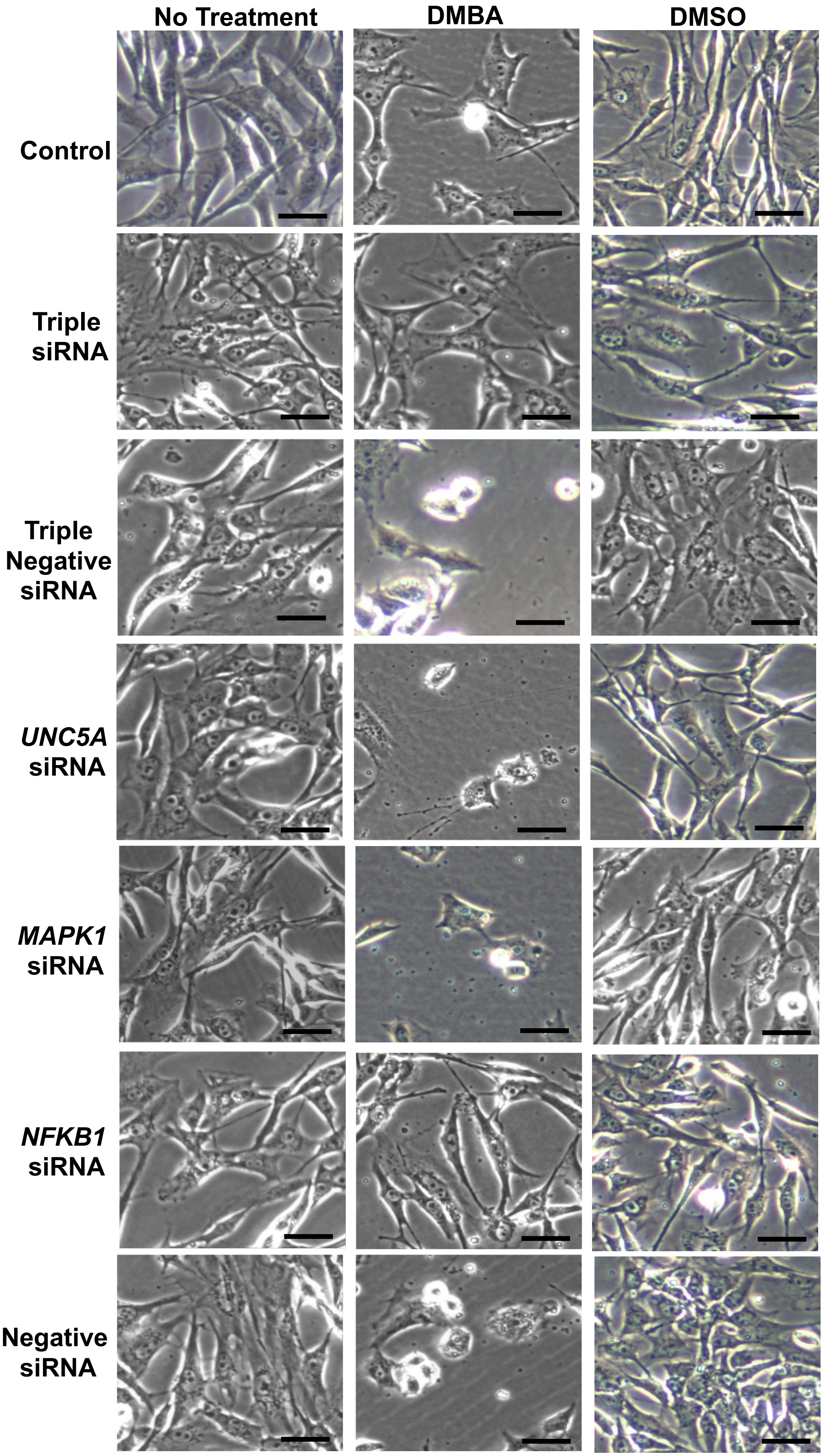

Supplementary Figure 4. Representative images of equine mammosphere-derived epithelial cells (MDECs) transfected with short-interfering RNAs (siRNAs) and treated with 7, 12-Dimethylbenz(a)anthracene (DMBA). Phase-contrast images of equine MDECs untransfected (control), transfected with a with a *UNC5A, MAPK1* and *NFKB1* siRNA cocktail or negative siRNA at the same concentration, or transfected with either a *UNC5A, MAPK1*, or *NFKB1* siRNA or negative siRNA. Transfections were followed by no treatment, treatment with 5 µM DMBA, or treatment with the vehicle control dimethlysulfoxide (DMSO). Scale bars = 20 µm.

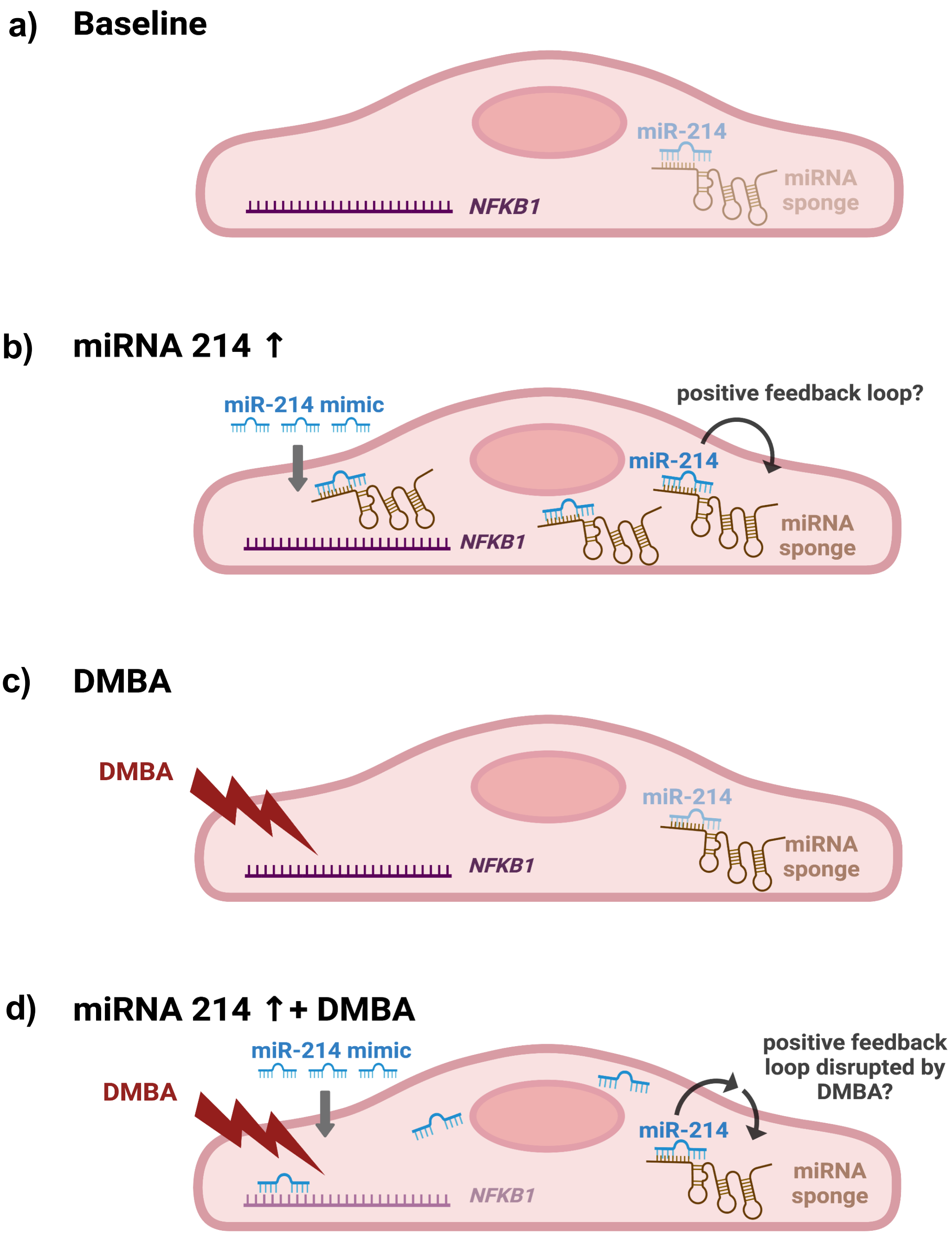

Supplementary Figure 5. A potential mechanism by which microRNA-214 (miR-214) and 7, 12-Dimethylbenz(a)anthracene (DMBA) decrease nuclear factor kappa beta 1 *(NFKB1)* expression in equine mammosphere derived epithelial cells (MDECs). a) At baseline, miR-214 expression is low, and a miRNA sponge blocks its function, allowing for robust expression of target *NFKB1*. **b)** Addition of a miR-214 mimic creates a positive feedback loop with increased expression of the miR-214 sponge, which binds to and blocks the activity of the supplementary miR-214. Expression of *NFKB1* continues to be robust as miR-214 activity is blocked. **c)** DMBA treatment alone does not affect *NFKB1* expression. **d)** When addition of a miR-214 mimic is followed by DMBA treatment, the positive feedback loop between miR-214 and the miR-214 sponge is disrupted by DMBA. miRNA-214 activity is no longer blocked and the excess of free miR-214 can interact with the 3'UTR of *NFKB1,* resulting in decreased *NFKB1* expression. Images created with BioRender.com.

Supplementary Table 1. Predicted miR-214-3p target genes.

CBX3
ANKRD52
ETS1
NPTN
INCENP
CDS2
ARVCF
EDF1
DIAPH1
KDM2A
MAPK1
TBC1D10B
CDK2
UGGT1
UNC5A
ATG16L1
TMEM161B
DEK
CDIPT
ZNF609
CAMK2G
NAA15
CPSF4
SLC25A39
RAB15
PLS3
TAF15
RAB5B
PARP16
CDC42BPB
ACVR1B
LARP1
MLXIP
YWHAZ
PLEKHG3
CAPRIN1
TSPAN9
BAHD1
ARPC5L
MYO18A
TMEM86A
LIPG
PPME1

PPP2CB
LIMK2
MMS19
IGF1R
STX6
CHPF
DOLPP1
PLA2G3
TRIP10
MAP1A
RAB34
GPR161
PNPLA6
CSF1
MARK2
CIT
USP30
CIB1
MAP4
ATP2A2
MAP3K4
DHX35
CAMSAP1
SEC24C
BAZ2A
GALNT7
PLD2
GANC
BRPF3
FBXL18
HDLBP
CTDSP1
LMOD1
USP42
SPCS2
ACLY
ESRRA
EIF4G1
GPN1
SORL1
PSAP
WDTC1
ARF4
PPP6C
CRKL

CTBP1
DMXL2
PSIP1
PTGS2
ERRFI1
ATP2B4
TMEM63A
MINK1
PIM1
SMYD5
NFKB1
RPIA
