# Supplementary material for: miRNA-214-3p stimulates carcinogen-induced mammary epithelial cell apoptosis in mammary cancer-resistant species
Source: Commun Biol. 2023 Oct 3;6:1006. doi: 10.1038/s42003-023-05370-4 (PMC10547694; doi:10.1038/s42003-023-05370-4)
Supplement: Supplementary file 3 — Description of Supplementary Materials [file 42003_2023_5370_MOESM3_ESM.docx]

**Description of Additional Supplementary Files**

**File name:** Supplementary Data 1

**Description:** Differentially expressed miRNAs.

**File name:** Supplementary Data 2

**Description:** Differentially expressed genes.

**File name**: Supplementary Data 3

**Description:** The source data behind the graphs in the paper.
