# Supplementary material for: miRNA-214-3p stimulates carcinogen-induced mammary epithelial cell apoptosis in mammary cancer-resistant species
Source: Commun Biol. 2023 Oct 3;6:1006. doi: 10.1038/s42003-023-05370-4 (PMC10547694; doi:10.1038/s42003-023-05370-4)
Supplement: Supplementary file 7 — reporting summary [file 42003_2023_5370_MOESM7_ESM.pdf]

Corresponding author(s): Van de Walle GR

Last updated by author(s): Sep 6, 2023

Reporting Summary

Nature Portfolio wishes to improve the reproducibility of the work that we publish. This form provides structure for consistency and transparency in reporting. For further information on Nature Portfolio policies, see our Editorial Policies and the Editorial Policy Checklist.

Statistics

For all statistical analyses, confirm that the following items are present in the figure legend, table legend, main text, or Methods section.

n/a	Confirmed	
☐	☒	The exact sample size (*n*) for each experimental group/condition, given as a discrete number and unit of measurement
☐	☒	A statement on whether measurements were taken from distinct samples or whether the same sample was measured repeatedly
☐	☒	The statistical test(s) used AND whether they are one- or two-sided<br>*Only common tests should be described solely by name; describe more complex techniques in the Methods section.*
☒	☐	A description of all covariates tested
☐	☒	A description of any assumptions or corrections, such as tests of normality and adjustment for multiple comparisons
☐	☒	A full description of the statistical parameters including central tendency (e.g. means) or other basic estimates (e.g. regression coefficient) AND variation (e.g. standard deviation) or associated estimates of uncertainty (e.g. confidence intervals)
☒	☐	For null hypothesis testing, the test statistic (e.g. *F*, *t*, *r*) with confidence intervals, effect sizes, degrees of freedom and *P* value noted<br>*Give P values as exact values whenever suitable.*
☒	☐	For Bayesian analysis, information on the choice of priors and Markov chain Monte Carlo settings
☒	☐	For hierarchical and complex designs, identification of the appropriate level for tests and full reporting of outcomes
☒	☐	Estimates of effect sizes (e.g. Cohen's *d*, Pearson's *r*), indicating how they were calculated

Our web collection on statistics for biologists contains articles on many of the points above.

Software and code

Policy information about availability of computer code

Data collection	*Provide a description of all commercial, open source and custom code used to collect the data in this study, specifying the version used OR state that no software was used.*
Data analysis	Sequencing reads were aligned to the Equus caballus reference genome (r2.9) using the STAR aligner (v2.4.2) and reads aligning to the transcriptome were quantified using Salmon (v0.8.2).

For manuscripts utilizing custom algorithms or software that are central to the research but not yet described in published literature, software must be made available to editors and reviewers. We strongly encourage code deposition in a community repository (e.g. GitHub). See the Nature Portfolio guidelines for submitting code & software for further information.

Data

Policy information about availability of data

All manuscripts must include a data availability statement. This statement should provide the following information, where applicable:

- Accession codes, unique identifiers, or web links for publicly available datasets
- A description of any restrictions on data availability
- For clinical datasets or third party data, please ensure that the statement adheres to our policy

The miRNA sequencing data are accessible through GEOSeries # GSE126424 (https://www.ncbi.nlm.nih.gov/geo/query/acc.cgi?acc=GSE126424). The RNA sequencing data are accessible through GEOSeries #GSE189879 (https://www.ncbi.nlm.nih.gov/geo/query/acc.cgi?acc=GSE189879).

Research involving human participants, their data, or biological material

Policy information about studies with human participants or human data. See also policy information about sex, gender (identity/presentation), and sexual orientation and race, ethnicity and racism.

Reporting on sex and gender	*Use the terms sex (biological attribute) and gender (shaped by social and cultural circumstances) carefully in order to avoid confusing both terms. Indicate if findings apply to only one sex or gender; describe whether sex and gender were considered in study design; whether sex and/or gender was determined based on self-reporting or assigned and methods used.*<br>*Provide in the source data disaggregated sex and gender data, where this information has been collected, and if consent has been obtained for sharing of individual-level data; provide overall numbers in this Reporting Summary. Please state if this information has not been collected.*<br>*Report sex- and gender-based analyses where performed, justify reasons for lack of sex- and gender-based analysis.*
Reporting on race, ethnicity, or other socially relevant groupings	*Please specify the socially constructed or socially relevant categorization variable(s) used in your manuscript and explain why they were used. Please note that such variables should not be used as proxies for other socially constructed/relevant variables (for example, race or ethnicity should not be used as a proxy for socioeconomic status).*<br>*Provide clear definitions of the relevant terms used, how they were provided (by the participants/respondents, the researchers, or third parties), and the method(s) used to classify people into the different categories (e.g. self-report, census or administrative data, social media data, etc.)*<br>*Please provide details about how you controlled for confounding variables in your analyses.*
Population characteristics	*Describe the covariate-relevant population characteristics of the human research participants (e.g. age, genotypic information, past and current diagnosis and treatment categories). If you filled out the behavioural & social sciences study design questions and have nothing to add here, write "See above."*
Recruitment	*Describe how participants were recruited. Outline any potential self-selection bias or other biases that may be present and how these are likely to impact results.*
Ethics oversight	*Identify the organization(s) that approved the study protocol.*

Note that full information on the approval of the study protocol must also be provided in the manuscript.

Field-specific reporting

Please select the one below that is the best fit for your research. If you are not sure, read the appropriate sections before making your selection.

☒ Life sciences ☐ Behavioural & social sciences ☐ Ecological, evolutionary & environmental sciences

For a reference copy of the document with all sections, see nature.com/documents/nr-reporting-summary-flat.pdf

Life sciences study design

All studies must disclose on these points even when the disclosure is negative.

Sample size	Each experiment consisted of 3 biological replicates (MDEC cell lines from 3 different animals), that were run in duplicate or triplicate, depending on the assay. Primary experiments (MTT assays comparing the responses of canine versus equine MDECs to DMBA and MTT assays comparing the responses of equine MDECs transfected with the miR-214 mimic versus controls to DMBA) were repeated 3 times.
Data exclusions	No data were excluded
Replication	All attempts at replication are presented in the data
Randomization	not applicable
Blinding	read-outs based on human observation (eg cell counts) were performed blinded.

Reporting for specific materials, systems and methods

We require information from authors about some types of materials, experimental systems and methods used in many studies. Here, indicate whether each material, system or method listed is relevant to your study. If you are not sure if a list item applies to your research, read the appropriate section before selecting a response.

Materials & experimental systems

n/a	Involved in the study
☐	☒ Antibodies
☐	☒ Eukaryotic cell lines
☒	☐ Palaeontology and archaeology
☒	☐ Animals and other organisms
☒	☐ Clinical data
☒	☐ Dual use research of concern
☒	☐ Plants

Methods

n/a	Involved in the study
☒	☐ ChIP-seq
☐	☒ Flow cytometry
☒	☐ MRI-based neuroimaging

Antibodies

Antibodies used	rabbit anti-active caspase-3 antibody (Abcam, ab4051, GR3296269-4); HRP-conjugated goat anti-rabbit secondary antibody (Jackson ImmunoResearch Labs); rabbit anti-UNC5A (LS Bio, C80729); rabbit anti-MAPK1 (LS Bio, C117080); rabbit anti-NF-kB (LS Bio, B8072); rabbit anti-b-actin (Abcam, ab8227); rabbit anti-IgG (Abcam, ab172730, clone EPR25A).
Validation	We previously published on using the rabbit anti-active caspase-3 antibody for our species of interest (i.e. horse): Ledet, M. M., Oswald, M., Anderson, R. & Van de Walle, G. R. Differential signaling pathway activation in 7,12-dimethylbenz[a] anthracene (DMBA)-treated mammary stem/progenitor cells from species with varying mammary cancer incidence. Oncotarget 9, 32761–32774 (2018). The antibodies against UNC5A, MAPK1, NF-kB, that we used for western blot analyses, had not been previously used for equine cells, but our positive controls indicated cross-reactivity .

Eukaryotic cell lines

Policy information about cell lines and Sex and Gender in Research

Cell line source(s)	Primary mammosphere-derived epithelial cell cultures were established from mammary gland tissues from euthanized healthy, non-lactating, research mares and beagles (female).<br>HeLa cells (female) were obtained through ATCC.
Authentication	Primary mammosphere-derived epithelial cell cultures have been characterized in previously published work: Spaas JH, Chiers K, Bussche L, Burvenich C, Van de Walle GR. Stem/progenitor cells in non-lactating versus lactating equine mammary gland. Stem Cells Dev. 2012 Nov 1;21(16):3055-67. doi: 10.1089/scd.2012.0042. Epub 2012 Jun 25. PMID: 22574831.<br>No authentication was done for HeLa cells as they were directyl obtained from ATCC.
Mycoplasma contamination	We check all our cell lines routinely for Mycoplasma contamination
Commonly misidentified lines<br>(See ICLAC register)	Not applicable

Flow Cytometry

Plots

Confirm that:

- ☐ The axis labels state the marker and fluorochrome used (e.g. CD4-FITC).
- ☐ The axis scales are clearly visible. Include numbers along axes only for bottom left plot of group (a 'group' is an analysis of identical markers).
- ☐ All plots are contour plots with outliers or pseudocolor plots.
- ☒ A numerical value for number of cells or percentage (with statistics) is provided.

Methodology

Sample preparation	Primary mammosphere-derived epithelial cell cultures were established from mammary gland tissues from euthanized healthy, non-lactating, research mares and beagles (female).
Instrument	BD Fortessa X-20 flow cytometer (BD Biosciences)
Software	data were analyzed using FlowJo software (Ashland, OR)
Cell population abundance	not applicable
Gating strategy	no gating

☐ Tick this box to confirm that a figure exemplifying the gating strategy is provided in the Supplementary Information.
